# An intracavitary right ventricular mass: a diagnostic dilemma—a case report

**DOI:** 10.1093/ehjcr/ytag676

**Published:** 2026-09-08

**Authors:** Jiries Haddad, Dhruva Govil, Sushmithaa Ramesh, Paul Weber, Saba Darda

**Affiliations:** Department of Internal Medicine, Henry Ford Providence, 16001 W Nine Mile Rd, Southfield, MI 48075, USA; Department of Internal Medicine, Henry Ford Providence, 16001 W Nine Mile Rd, Southfield, MI 48075, USA; Department of Internal Medicine, Henry Ford Providence, 16001 W Nine Mile Rd, Southfield, MI 48075, USA; Department of Cardiology, Henry Ford Providence, 16001 W Nine Mile Rd, Southfield, MI 48075, USA; Department of Cardiology, Henry Ford Providence, 16001 W Nine Mile Rd, Southfield, MI 48075, USA

**Keywords:** Non-small cell lung cancer, Cardiac metastasis, Cardiac magnetic resonance, Right ventricular outflow tract, Case report

## Abstract

**Background:**

Intracardiac masses are a diagnostic challenge in cardiology because thrombus and malignancy may demonstrate similar imaging features on initial evaluation.

**Case summary:**

An elderly male admitted for sepsis was incidentally found to have a right ventricular outflow tract mass initially presumed to be thrombus, and anticoagulation was initiated. Follow-up imaging demonstrated interval enlargement despite therapy, prompting further evaluation. Transesophageal echocardiography and cardiac magnetic resonance raised concern for malignancy, and biopsy confirmed poorly differentiated non–small cell lung carcinoma with cardiac metastasis. Given advanced disease and poor functional status, disease-directed therapy was deferred, and hospice care was pursued.

**Discussion:**

Cardiac metastases are more common than primary cardiac tumours and occur in up to 9%–14% of patients with metastatic cancer, with lung cancer representing the most frequent primary source. Prior literature emphasizes multimodal imaging when presumed thrombus fails to respond to anticoagulation.

**Conclusion:**

Interval growth of an intracardiac mass despite anticoagulation should prompt diagnostic reassessment and advanced cardiac imaging.

Learning points

- Intracardiac masses that persist or enlarge despite therapeutic anticoagulation should prompt re-evaluation for malignancy.
- Multimodality imaging, especially CMR, is essential for differentiating thrombus from tumours when initial imaging is inconclusive.

## Introduction

Intracardiac masses present a diagnostic challenge because thrombi, primary cardiac tumours, and metastatic lesions may have overlapping features on initial imaging. Cardiac metastases are more common than primary cardiac malignancies and are identified in up to 14% of patients with advanced metastatic cancer, with lung cancer representing the most frequent primary source. However, endocardial or intracavitary metastases are uncommon, accounting for fewer than 3% of cases.^1^

Distinguishing thrombus from malignancy requires integration of lesion morphology, location, clinical context, and response to therapy. When a presumed thrombus persists or enlarges despite anticoagulation, the diagnosis should be reconsidered and advanced imaging pursued. We present a case of an enlarging right ventricular outflow tract mass initially treated as thrombus and ultimately identified as metastatic non-small cell lung carcinoma.

## Summary figure

**Figure:**
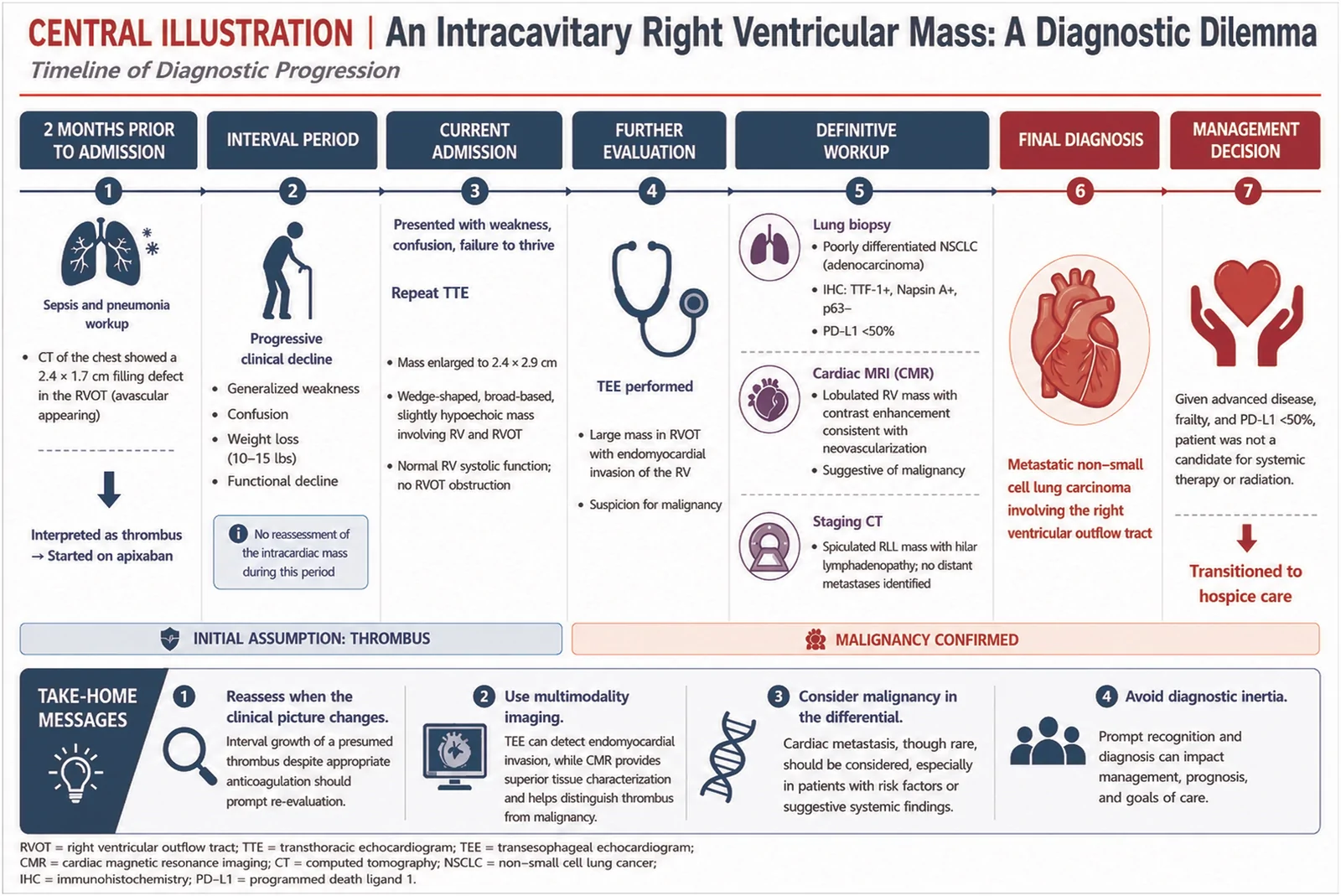


## Case description

**Figure 1:**
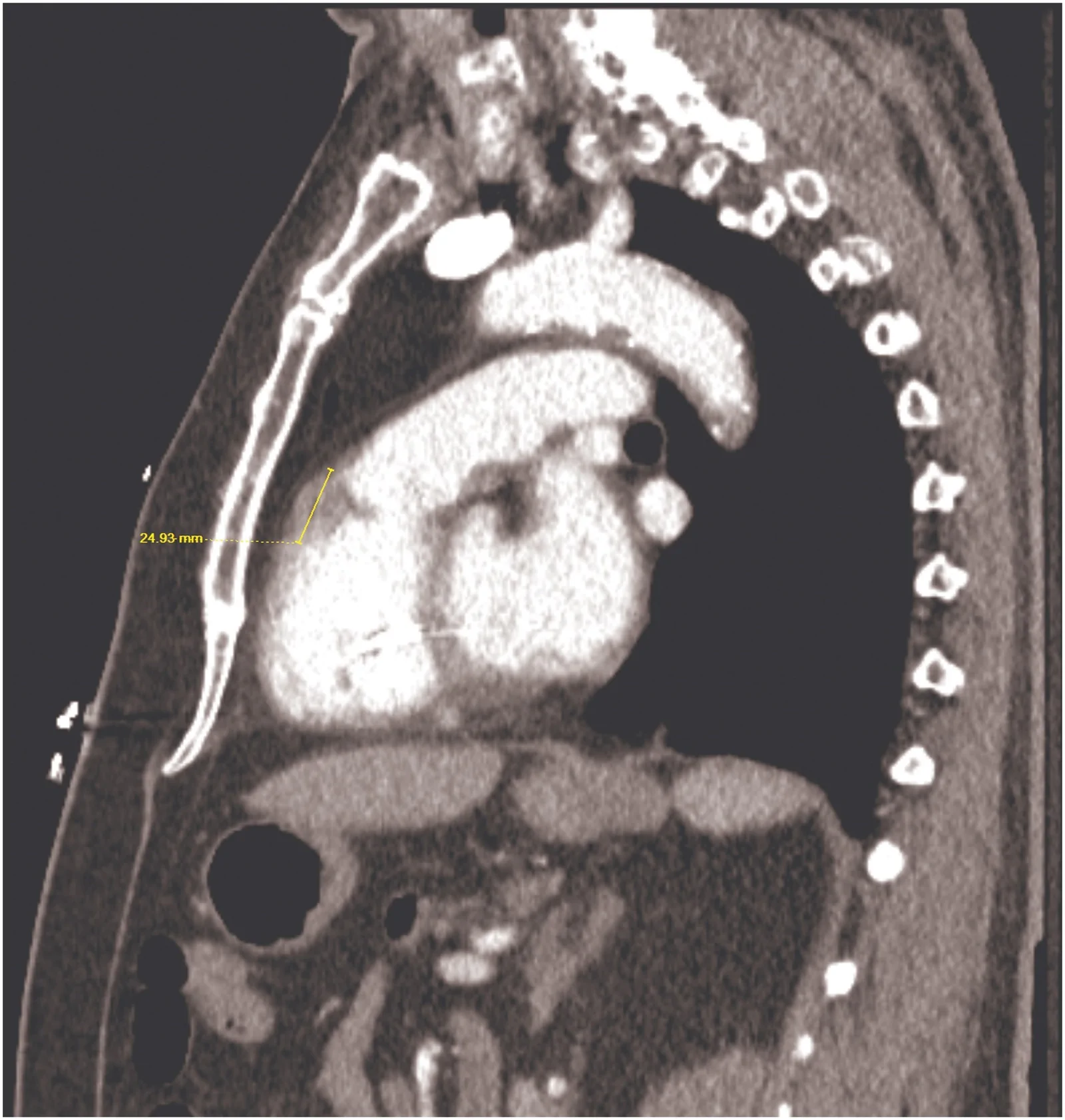
Computed tomography of the chest, sagittal plane. Hypodense filling defect is identified within the right ventricular outflow tract, measuring ∼25 mm in greatest dimension.

A 79-year-old male presented with progressive generalized weakness, worsening confusion, functional decline, and unintentional 10–15 lb weight loss. On examination, he was bedbound and oriented only to person and place. Two months earlier, during hospitalization for pneumonia and sepsis, computed tomography (CT) of the chest and transthoracic echocardiography (TTE) identified a 2.4 × 1.7 cm avascular filling defect within the right ventricular outflow tract (RVOT) (*Figure 1*), initially presumed to represent thrombus, and anticoagulation with apixaban was initiated. Given the patient’s concurrent hospitalization for pneumonia and sepsis, the absence of a known primary malignancy, and the initial interpretation of an avascular filling defect on CT and TTE, the lesion was considered most consistent with thrombus. Accordingly, anticoagulation was initiated, and advanced tissue characterization with cardiac magnetic resonance imaging (CMR) was deferred during the initial hospitalization pending assessment of interval response to therapy.

His medical history was significant for coronary artery disease status post percutaneous coronary intervention to the left circumflex artery, hypertension, Barrett’s oesophagus, prostate cancer status post radical prostatectomy and radiation therapy, permanent pacemaker placement for complete heart block, dementia, and prior tobacco use.

**Figure 2:**
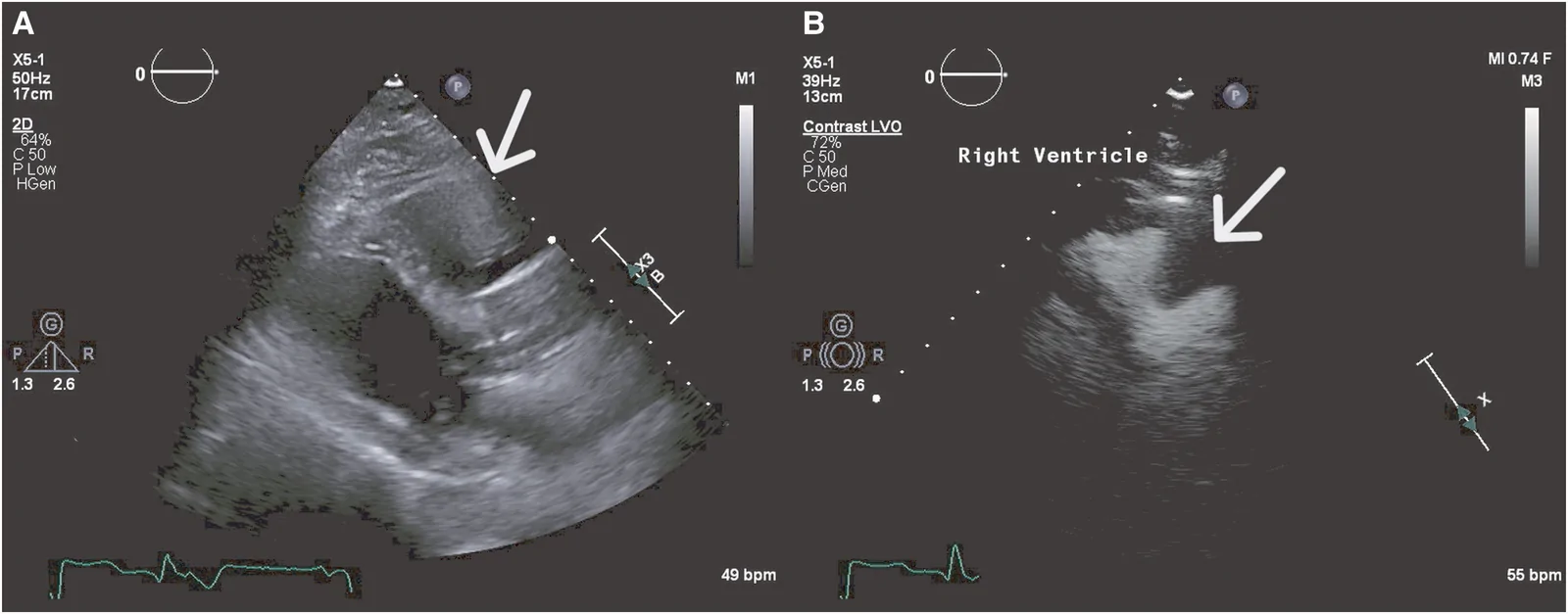
Transthoracic echocardiogram parasternal view. (*A*) Demonstration of the right ventricular mass measuring ∼2.4 × 2.9 cm, described as a wedge-shaped, broad-based, slightly hypoechoic mass involving the right ventricle and right ventricular outflow tract (indicated by white arrow). (*B*) Contrast-enhanced imaging demonstrates no contrast uptake within the mass (indicated by white arrow).

**Figure 3:**
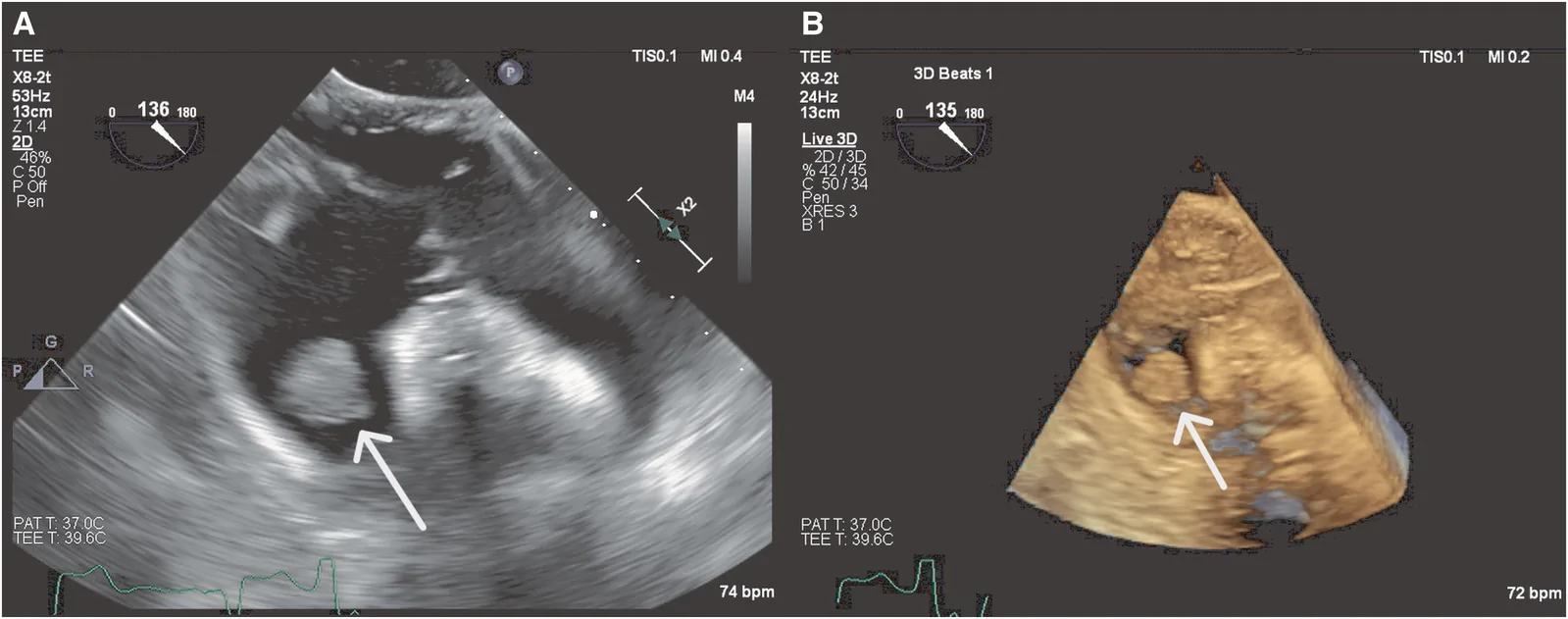
Transoesophageal echocardiography midesophageal views. (*A*) At 136°, a large echogenic mass measuring ∼3.0 × 2.4 cm is seen within the right ventricular outflow tract, along the right ventricular free wall in the subpulmonic region (white arrow). (*B*) A 3-dimensional view of the mass demonstrating endomyocardial invasion at the right ventricular free wall (white arrow).

Repeat TTE demonstrated interval enlargement of the mass to ∼2.4 × 2.9 cm, described as a wedge-shaped, broad-based hypoechoic lesion involving the right ventricle and RVOT without evidence of RVOT obstruction (*Figure 2*). Transoesophageal echocardiography (TEE) further characterized a large echogenic mass measuring ∼3.0 × 2.4 cm along the right ventricular free wall in the subpulmonic region with invasion into the endomyocardial border (*Figure 3*). Differential diagnosis included right ventricular thrombus, primary cardiac malignancy, and metastatic cardiac disease.

**Figure 4:**
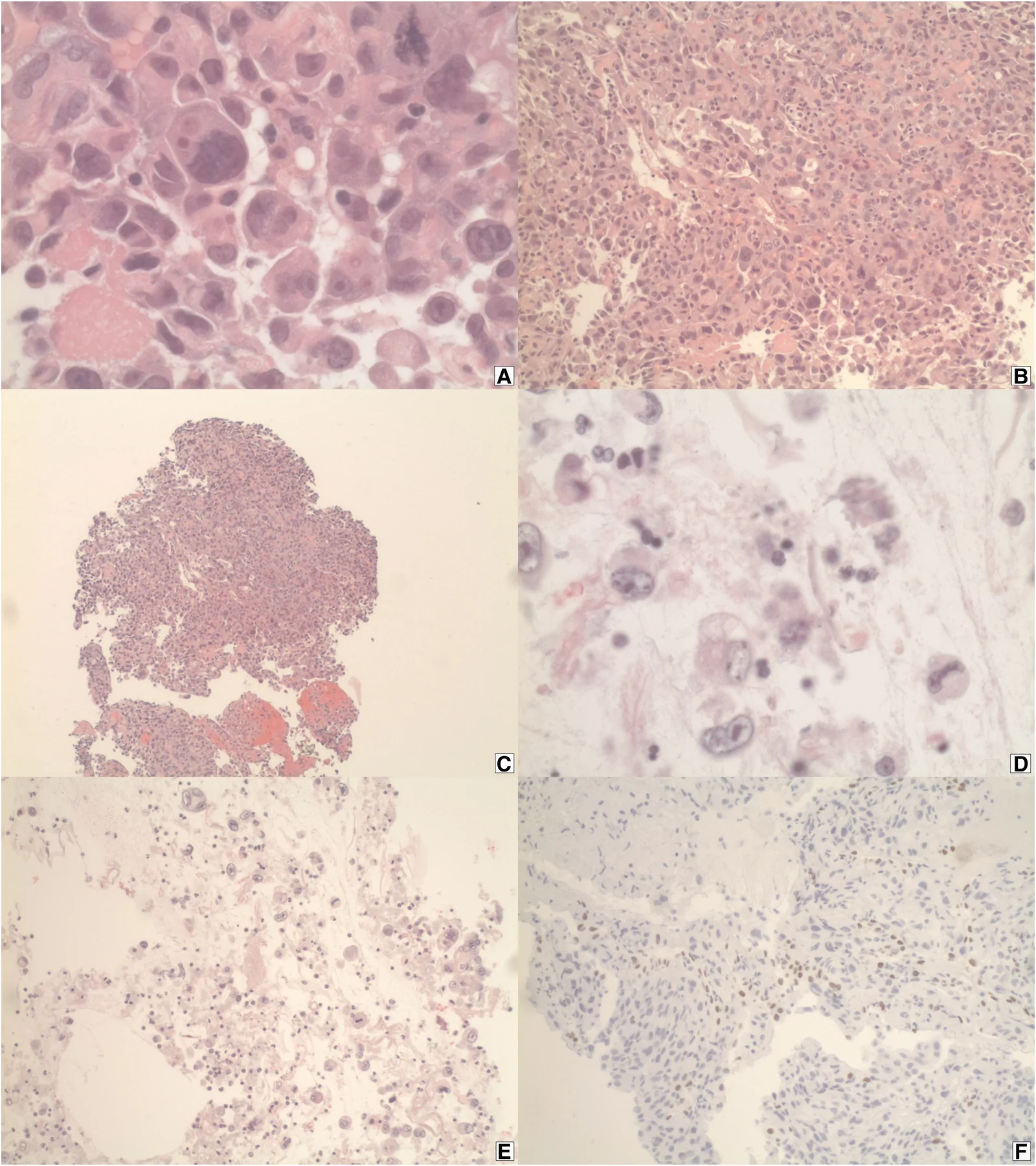
Histopathologic evaluation of the right lower lobe lung lesion. (*A*) High-power haematoxylin and eosin stain (×40) demonstrates severe nuclear pleomorphism with vesicular chromatin, prominent nucleoli, abundant eosinophilic cytoplasm, and a mitotic figure. (*B*) Intermediate-power haematoxylin and eosin stain (×10) showing a solid growth pattern without identifiable acinar, papillary, or lepidic architecture. (*C*) Low-power haematoxylin and eosin stain (×4) illustrating the overall tissue architecture of a poorly differentiated non-small cell carcinoma, favouring adenocarcinoma. (*D*) Fine needle aspiration cytology, high-power view (×40), demonstrates large malignant cells with abundant cytoplasm, vesicular nuclei, and prominent nucleoli. (*E*) Intermediate-power fine needle aspiration cytology (×10) showing high cellularity and clustered malignant cells. (*F*) Immunohistochemical staining of the right lower lobe core biopsy demonstrates diffuse nuclear positivity for thyroid transcription factor-1 and cytoplasmic positivity for Napsin A, supporting a primary lung adenocarcinoma, while p63 is negative, arguing against squamous cell carcinoma.

**Figure 5:**
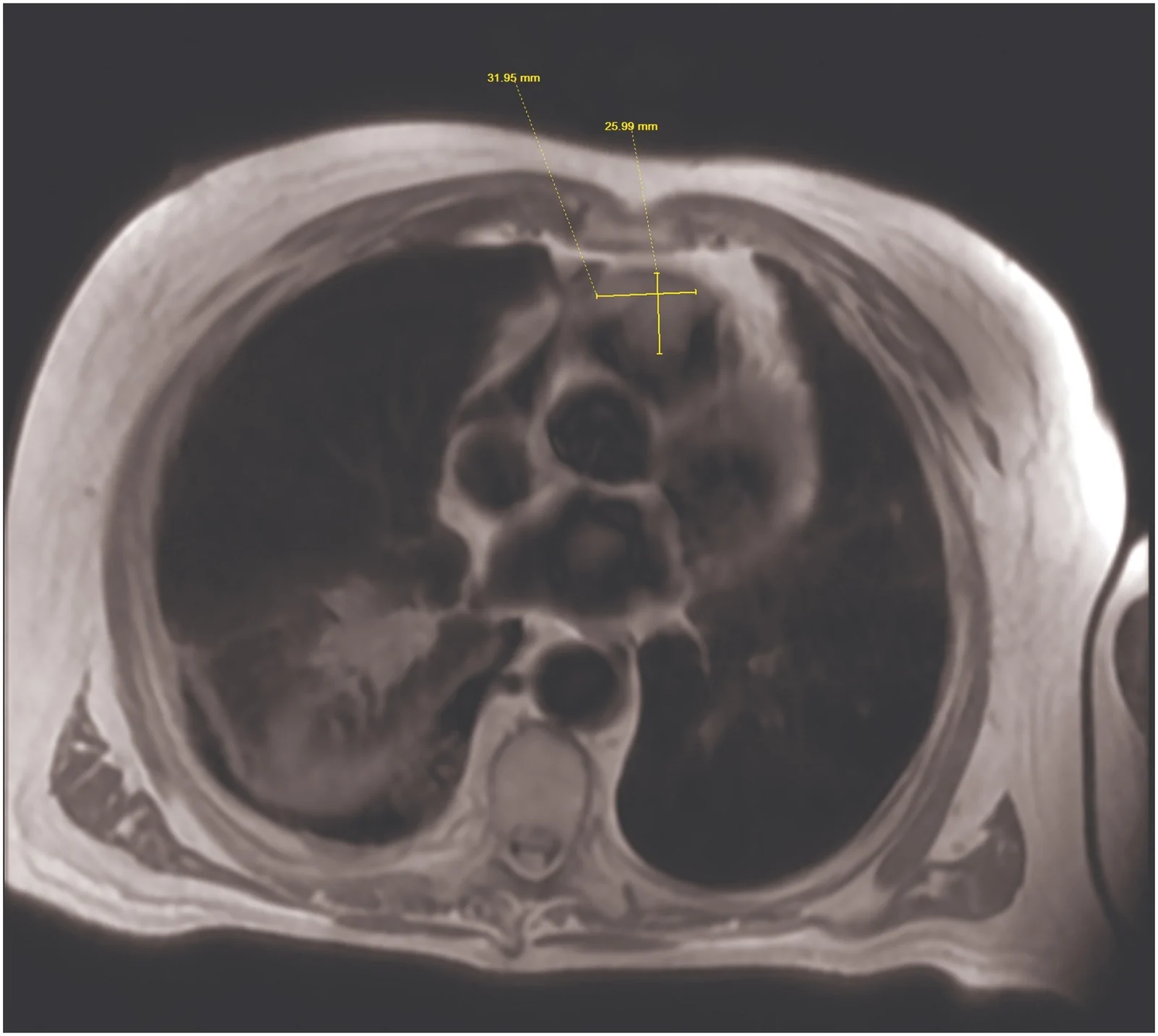
Cardiac magnetic resonance imaging. Demonstrating a lobulated mass (arrows) along the anterior right ventricular free wall, extending into the right ventricular lumen proximal to the pulmonic valve.

Endobronchial ultrasound-guided biopsy of a right lower lobe lesion demonstrated poorly differentiated non-small cell lung carcinoma favouring adenocarcinoma, with immunohistochemical positivity for TTF-1 and Napsin A and negative p63 staining (*Figure 4*). Subcarinal lymph node biopsy was negative for malignancy. Computed tomography of the head, abdomen, and pelvis showed no additional extracardiac metastatic disease. Cardiac magnetic resonance imaging demonstrated a lobulated mass involving the anterior right ventricular wall protruding into the lumen proximal to the pulmonic valve, with imaging characteristics most consistent with metastatic cardiac involvement rather than primary cardiac malignancy (*Figure 5*).

Given PD-L1 expression below 50%, combination chemoimmunotherapy would have been required; however, the patient was not considered a candidate for systemic therapy, radiation, or surgical intervention because of severe frailty and poor functional status. Following multidisciplinary discussions and shared decision-making with the patient and family, care was transitioned to comfort-focused hospice management.

## Discussion

When an intracardiac mass is identified, thrombus remains an important initial consideration, particularly in patients with systemic illness or intracardiac devices. Current guidelines recommend systemic anticoagulation for confirmed left ventricular thrombus for at least 3 months, followed by repeat imaging to assess resolution.^2^ In this case, initiation of anticoagulation was appropriate based on the initial imaging interpretation.^2^ Contrast echocardiographic perfusion imaging can further aid in differentiating thrombus from tumour. Thrombi typically demonstrate complete absence of contrast enhancement due to their avascular structure, whereas malignant tumours commonly show hyperenhancement reflecting neovascularization.^3^ However, interval enlargement of the mass despite therapy prompted reconsideration of thrombus as the underlying diagnosis and led to further evaluation through multimodality imaging.

In retrospect, although thrombus remained a reasonable initial consideration, several imaging and clinical features made the diagnosis less straightforward. The lesion demonstrated a broad-based attachment to the right ventricular free wall and RVOT without evidence of regional wall motion abnormalities or significant right ventricular dysfunction. While these findings were not diagnostic of malignancy, they were atypical for thrombus and warranted earlier consideration of alternative diagnoses. This case highlights the importance of integrating lesion morphology with the overall clinical context and maintaining a low threshold for advanced multimodality imaging, particularly CMR, when the imaging appearance or clinical course is not entirely consistent with thrombus.^2,4–6^ This case illustrates the clinical consequences of diagnostic inertia; the initial assumption of a thrombus led to a 2-month delay in recognizing a Stage IV malignancy. While lung cancer is the most common primary source of cardiac metastases, endocardial lesions occur in only 3% to 5% of cases and are hallmark indicators of advanced haematogenous spread.^7^

The right ventricle is a recognized site of cardiac metastatic involvement where intracavitary lesions may mimic the appearance of right ventricular thrombus.^1,4^ Although pulmonary adenocarcinoma metastasizing to the right ventricle with both intracavitary and endomyocardial involvement has rarely been described, reports of RVOT metastatic disease remain uncommon.^4,8–10^ Published reports have demonstrated that these lesions may initially be mistaken for thrombus on echocardiography, with definitive diagnosis often requiring multimodality imaging and histopathologic confirmation.^8–10^ Notably, the pericardium remains the most common site of cardiac metastatic involvement, accounting for ∼64%–69% of cases in large series.^4^ The absence of a pericardial effusion in our patient further contributed to the initial diagnostic uncertainty. Our case reinforces these observations while highlighting the additional diagnostic challenge posed by an isolated RVOT lesion with endomyocardial involvement in the absence of regional wall motion abnormalities. These findings emphasize the importance of reconsidering malignancy when intracardiac masses demonstrate atypical imaging features or fail to respond as expected to anticoagulation.^2,6^

Multimodality imaging plays a central role in differentiation. TTE serves as the first-line imaging modality for evaluation of suspected intracardiac masses, providing assessment of size, mobility, attachment, and haemodynamic impact. When TTE findings are inconclusive, TEE offers improved spatial resolution and visualization of intracardiac structures.^5^ In this patient, TEE was appropriately pursued after interval enlargement of the presumed thrombus raised concern for an alternative aetiology. CMR further improved diagnostic confidence through superior tissue characterization. CMR offers superior tissue characterization and diagnostic accuracy, reported at ∼96% for cardiac tumours, and is particularly useful in differentiating thrombus, which typically lacks contrast enhancement, from tumours, which more commonly demonstrate enhancement and infiltrative features.^6^ In this patient, CMR first-pass perfusion imaging demonstrated early gadolinium uptake through the mass, a hallmark of neovascularization in malignant tissue, whereas a thrombus would remain a dark or signal void of contrast due to its inherent lack of a capillary bed.

Cardiac metastasis carries significant prognostic implications and generally reflects advanced systemic disease associated with poor outcomes.^11^ Cardiac metastases are most often identified in the setting of disseminated malignancy and are generally associated with widespread metastatic disease.^4,11^ Among patients with cardiac metastases from lung cancer, reported median survival is poor.^12^ In this case, histopathologic evaluation confirmed lung adenocarcinoma with PD-L1 expression <50%. For inoperable disease, prognosis is severely impacted by poor performance status and weight loss exceeding 10%; these factors effectively excluded him from aggressive multimodality interventions.^12^ In the absence of viable therapeutic options and given the patient’s frailty and overall prognosis, transition to hospice care represented a goal-concordant and medically appropriate management decision.

## Conclusion

This case underscores the diagnostic challenge of distinguishing intracardiac thrombus from cardiac malignancy when a right ventricular mass is identified. Although initial management with anticoagulation was appropriate based on imaging interpretation, lack of expected therapeutic response should prompt reassessment. Multimodality imaging, particularly CMR, is critical for accurate tissue characterization. Early reconsideration of the diagnosis may prevent delays in identifying underlying malignancy and guide appropriate management.

## Lead author biography


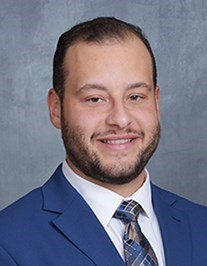


Dr Jiries Haddad is an Internal Medicine resident physician at Henry Ford Providence in Michigan with a strong interest in cardiovascular medicine, multimodality cardiac imaging, and complex diagnostic cases. He earned his medical degree at William Carey College of Osteopathic Medicine. His academic interests include general cardiology, heart failure, cardiac masses, and cardiac intervention. He plans to pursue fellowship training in cardiology and remains actively involved in scholarly work and medical education.

## Supplementary Material

ytag676_Supplementary_Data

## Data Availability

The data underlying this article are available from the corresponding author upon reasonable request.
